# Associations between gastric cancer risk and virus infection other than Epstein-Barr virus: The protocol of a systematic review and meta-analysis based on epidemiological studies

**DOI:** 10.1097/MD.0000000000016708

**Published:** 2019-08-09

**Authors:** Rui Wang, Kai Liu, Xin-Zu Chen

**Affiliations:** a Nursing Section, Department of Gastroenterology; b Department of Gastrointestinal Surgery and Laboratory of Gastric Cancer, West China Hospital, Sichuan University, Chengdu, China.

**Keywords:** epidemiology, etiology, gastric cancer, infection, serology, virus

## Abstract

**Background:**

Gastric cancer is one of the infection associated malignancies. In addition to Helicobacter pylori and Epstein-Barr virus (EBV), other oncoviruses might play potential roles in the development of gastric cancer. Associations of oncoviruses other than EBV with gastric cancer risk are aimed to be comprehensively reviewed and assessed in this systematic review and meta-analysis, to identify any potentially causative oncovirus. It might be informative to identify or deny certain oncoviruses which are candidates of risk factor for gastric cancer. To our knowledge, there is no comprehensive review on oncoviruses other than EBV associated with gastric cancer risk. Positive findings might be helpful to suggest further mechanism investigation and high-risk subpopulation recommendation.

**Methods:**

PubMed database will be searched up to Dec 31, 2018. The studies, compared the positivity of any oncovirus other than EBV between cases with histologically proven gastric cancer and healthy or nonmalignant controls, are eligible. The detection of oncovirus either in tissue or blood is acceptable. Selection, quality assessment (Newcastle-Ottawa Scale), and data extraction of eligible studies will be performed by 2 independent reviewers. Pooled prevalence of any oncovirus will be combined by meta-analysis for rate. Pooled odds ratio between gastric cancer cases and controls will be estimated by meta-analysis. Heterogeneity and publication bias will be tested. In sensitivity analysis, the leave-one-out method and exclusion of low power studies will be applied where applicable.

**Results:**

This review was not submitted for any ethical approval due to the literature-based nature. The results will be published in a journal and presented at conferences for academic purposes.

Registration number was CRD42015029703 in the PROSPERO International Prospective Register of Systematic Reviews.

**Conclusions:**

To our knowledge, there is no comprehensive review on oncoviruses other than EBV associated with gastric cancer risk. Positive findings might be helpful to suggest further mechanism investigation and high-risk subpopulation recommendation.

**Key Points**

- To our knowledge, there is no comprehensive review on oncoviruses other than EBV associated with gastric cancer risk.
- Positive findings might be helpful to suggest further mechanism investigation and high-risk subpopulation recommendation.
- The major difficulty of this systematic review might be the lack of relevant researches on this topic.
- Meta-analysis might be impossible due to limited published data, or diverse detection methods.
- Despite of any positive association, the causative function of the specific oncovirus may still not be evidenced due to small-scaled observations, or complex and underestimated confounders.

## 1 Introduction

Gastric cancer is one of the leading mortality malignancies worldwide,^[1]^ and caused 782,685 deaths according to the GLOBOCAN 2018 fact sheet.^[2]^ The 5-year overall survival of gastric cancer is fairly poor worldwide,^[3]^ since most countries do not establish a nationwide gastric cancer screening program for the high-risk subpopulation, except Japan and Korea.^[4–6]^ The difficulty in early detection and treatment at early stage is still a comprehensive issue to improve population survival outcome of gastric cancer.^[7,8]^ Currently, it keeps no definitely efficient biomarker or combinations to identify gastric cancer cases for the sake of the early detection.^[9,10]^ Therefore, the necessity of identifying exposure factors associated with the gastric cancer risk is underlined. It may be helpful to organize the screening, prevention, and surveillance program for the high-risk subpopulation of gastric cancer.

As the World Health Organization suggested, gastric cancer is one of the infection associated malignancies, and therefore it could potentially be prevented and early detected among high-risk subpopulation.^[11–13]^ Helicobacter pylori (Hp) has been regarded as a biological risk factor for the carcinogenesis of gastric epithelium,^[14,15]^ especially highly associated with non-cardia gastric adenocarcinoma.^[16,17]^ Epidemiological data support Hp as the most important biological risk factor for gastric cancer in both Western and Eastern countries.^[18–20]^ Epstein-Barr virus (EBV) is very common among general population, and has also been known to be present in a small proportion of gastric cancer tissues.^[21]^ Some meta-analyses have concluded that EBV-positive gastric cancer might appear in 6.9% to 8.8% of all gastric cancer patients.^[22–25]^

Therefore, it is questionable whether any other oncovirus other than EBV would be associated with human gastric cancer risk. We will perform a systematic review and meta-analysis to estimate the associations of human gastric cancer risk with viral infection, in addition to EBV, based on epidemiological studies.

## 2 Methods

### 2.1 Search strategy

The PubMed database will be searched up to Dec 31, 2018, using the following search algorithm ((“stomach neoplasms”[MeSH Terms] OR (“stomach”[All Fields] AND “neoplasms”[All Fields]) OR “stomach neoplasms”[All Fields] OR (“gastric”[All Fields] AND “cancer”[All Fields]) OR “gastric cancer”[All Fields]) AND (“viruses”[MeSH Terms] OR “viruses”[All Fields] OR “virus”[All Fields])) NOT (“animal”[Filter] OR “review”[Publication Type] OR “case reports”[Publication Type] OR “letter”[Publication Type] OR “comment”[Publication Type]). The search was limited to studies in humans. There is no limitation of publication language.

### 2.2 Eligibility

The eligible studies compared the positivity of any oncovirus between cases with histologically proven gastric cancer to healthy or nonmalignant controls. The patients with gastric lymphoma, gastrointestinal stromal tumor, and squamous cell carcinoma are ineligible. Any type of cohort study, cross-sectional study, or case-control study can be considered. The detection of viruses was performed in cases and controls based on either blood/serum or tissue specimens. Any targeted virus other than EBV are eligible in this systematic review. With the only particular exception, EBV was not additionally considered in this systematic review, because it had been comprehensively assessed yet in the our previous study through the similar methodology as the first part of serial reviews.^[21]^ There is no limitation on the race, sex, age, cancer stage, and the treatment strategy. Only those studies with extractable data will be considered for analysis.

### 2.3 Selection and quality assessment

Two reviewers will separately browse the titles/abstracts, and assess the potentially eligible full-texts, according to the predefined inclusion and exclusion criteria. Discrepancies will be resolved by consensus with a third reviewer. Risk of bias assessment of all included studies is to be independently performed by 2 reviewers, according to the Newcastle-Ottawa Scale (NOS).^[26]^ The scale contains 8 criteria of 3 categories to evaluate the sample selection, comparability on the bases of design or analysis, outcome assessment.

### 2.4 Data extraction

Extracted items includes general study characteristics (year, country, study design), characteristics of the study population (size, sex, age, disease related factors), and types of measurements (specimen types, analytic procedures). Number of cases and controls will be extracted from publications or calculated from the reported percentage of cases. Potential discrepancies in extracted items if any will be resolved by further review and discussion.

### 2.5 Statistics

The Cochrane Reviewer Manager (RevMan) 5.3 (Copenhagen, Denmark), the STATA 12.0 softwares (Texas), and the PASS 11 (Utah) will be used for statistical analysis, where applicable.^[27–29]^ All analysis will be based on individual oncovirus. If quantitative combination is impossible, narrative review will be considered. The pooled prevalence of any oncovirus infection in gastric cancer patients and healthy controls will be combined in meta-analysis for rate, with 95% confidence intervals (CIs). The pooled odds ratios (ORs) and their 95% CIs for oncovirus prevalence will be calculated between gastric cancer patients and healthy controls by fixed or random effect model where suitable. The Mantel-Haenszel test or the DerSimonian-Laird test is to be used for fixed or random model respectively. Two-sided *P* values for the pooled ORs <0.05 is considered as statistical significance. I-square will be estimated to evaluate the heterogeneity of meta-analyses. If the *P* values of heterogeneity test <.1, random effect model should be considered. Funnel plots will be drawn by the STATA 12.0 software to evaluate the publication bias.^[28]^ Both the continuity corrected Begg rank correlation test and Egger linear regression test will be used.^[30]^ Any *P* value <.05 of Begg or Egger test is considered as significance of publication bias. In Egger test, the intercept and its 95% CI will be estimated. In sensitivity analysis, the leave-one-out method will be applied for those meta-analyses pooling at least 2 studies. Additionally, L’Abbé plot and Galbraith plot will be observed the heterogeneity. For an individual study, the power (1–β) will be estimated by the PASS 11 software.^[29]^ The category of 2 independent proportions to test inequality is selected, and parameter module of proportions is used for calculation.^[31]^ Two-sided Z test (pooled) is provided with *α* = 0.05. Additional sensitivity analysis will be performed by excluding the studies with the power <0.70 or <0.80.

### 2.6 Registration

The present systematic review (registration number: CRD42015029703) was registered in the PROSPERO International Prospective Register of Systematic Reviews supported by the National Institute for Health Research of the National Health Service (NHS), UK.^[32]^

### 2.7 Patient and public involvement

This review worked with the literature and will not directly involve human beings or animals. This study is a joint with the Sichuan Gastric Cancer Early Detection and Screening (SIGES) research project.^[33]^

### 2.8 Ethics and dissemination

The protocol was not submitted for any ethical approval due to the literature-based nature. The results of this study will be published in a peer-reviewed academic journal and presented at academic conferences.

### 2.9 Reporting

This meta-analysis were conducted according to the MOOSE 2000 statements,^[34]^ and a flow diagram is to be plotted.^[35]^

## 3 Discussion

Gastric cancer has been documented that is associated with the infection of Hp or EBV. Till now, Hp infected persons are suggested as high-risk subpopulation of gastric cancer development.^[13,20]^ In Japanese and Korean gastric cancer control programme, massive screening and eradication of Hp infection is organized and integrated into early gastric cancer detection.^[36–39]^ Early detection and eradication of Hp might decrease the incidence of gastric cancer,^[40]^ rather than in the stages of mucosal precancerous lesions.^[41]^ This is a successful attempt of a pathogen based gastric cancer control. Although the causative association was highly considered between gastric cancer risk and EBV infection,^[21]^ a plan of EBV based gastric cancer control has not been established yet. Moreover, in the cancer genome atlas investigation, EBV-positive gastric cancer has been clearly identified as a specific type of molecular classification.^[1]^ Therefore, pathogen guided gastric cancer research on mechanism or population screening, prevention and treatment would be a potentially important opportunity.

Besides, several other oncoviruses are considered to be associated with human cancers.^[42]^ For instance, some high-risk, oncogenic types of human papillomavirus (HPV) are able to result in cervical cancer, as well as potentially anal and colorectal cancer.^[43–45]^ In the 2009, the WHO recommended the HPV vaccination targeting young girls before the onset of sexual activity as a national immunization programs.^[46]^ At the early of the 2014, 21 European countries have included HPV vaccination for girls in their national immunization schedules, and 11 out of those countries have also introduced catch-up programs.^[47]^ Besides, a cost-effectiveness analysis in Denmark found the inclusion of both boys and girls is a cost-effective preventive approach to control cancers and cancer precursors in men and women.^[48]^ The potential association between HPV and gastric cancer risk would be interesting to studied in the present systematic review.

Similarly, hepatitis B virus (HBV) has been classified a causative pathogen for primary liver cancer, which had high incidence and mortality in China.^[49]^ Certain estimate suggested 60% to 80% primary liver cancers were the consequence of HBV infection and related chronic hepatitis.^[50]^ Therefore, since the 1992, massive newborn and pediatric HBV vaccination programme was started.^[6]^ Along with the decrease of HBV prevalence nationwide and expectation of below 1% according to the governmental estimate,^[51]^ the incidence and mortality of primary liver cancer would be hoped to controlled correspondingly. Interestingly, a case-control study found a potential association between HBV seropositivity (hepatitis B surface antigen) and gastric cancer risk, specially among those without family history of gastric cancer.^[52]^ However, the association was quite challenging, as the positivity of HBV DNA in gastric cancer tissues was only 0% to 3% and no direction evidence of HBV DNA detected in gastric cancer cells.^[53]^ This present systematic review will pay attention to the association between HBV and gastric cancer risk.

In addition, there are some other oncoviruses, such as HCV, JCV, CMV, BKV, SV40, HSV, and VZV, etc, will also be considered for evaluation, if possible. However, the major difficulty of this systematic review might be the lack of relevant researches on this topic. Meta-analysis might be impossible due to limited published data, or diverse detection methods. Despite of any positive association, the causative function of the specific oncovirus may still not be evidenced due to small-scaled observations, or complex and underestimated confounders.

In a short, this systematic review and meta-analysis might be informative to identify or deny certain oncoviruses which are candidates of risk factor for gastric cancer. To our knowledge, there is currently no comprehensive review on oncoviruses other than EBV associated with gastric cancer risk. Positive findings might be helpful to suggest further mechanism investigation and high-risk subpopulation recommendation.

## Acknowledgments

The work is a joint with the Sichuan Gastric Cancer Early Detection and Screening (SIGES) research project. The authors thank the substantial work of Volunteer Team of Gastric Cancer Surgery (VOLTGA), West China Hospital, Sichuan University, China.

## Author contributions

**Conceptualization**: Xin-Zu Chen.

**Data curation:** Rui Wang, Xin-Zu Chen.

**Formal analysis**: Xin-Zu Chen.

**Funding acquisition**: Xin-Zu Chen.

**Investigation:** Rui Wang, Kai Liu, Xin-Zu Chen.

**Methodology:** Xin-Zu Chen.

**Project administration**: Rui Wang, Kai Liu.

**Resources:** Xin-Zu Chen.

**Supervision:** Xin-Zu Chen.

**Validation:** Rui Wang, Kai Liu, Xin-Zu Chen.

**Writing – original draft**: Xin-Zu Chen.

**Writing – review & editing**: Xin-Zu Chen.

Rui Wang orcid: 0000-0003-0101-4431.

Kai Liu orcid: 0000-0002-7584-9231.

Xin-Zu Chen orcid: 0000-0002-7619-6244.
